# Long-Term Evolution of Quality of Life and Symptoms Following Surgical Treatment for Endometriosis: Different Trajectories for Which Patients?

**DOI:** 10.3390/jcm9082461

**Published:** 2020-07-31

**Authors:** Aurélie Comptour, Céline Lambert, Pauline Chauvet, Claire Figuier, Anne-Sophie Gremeau, Michel Canis, Bruno Pereira, Nicolas Bourdel

**Affiliations:** 1 CHU Clermont-Ferrand, Service de Chirurgie Gynécologique, 63000 Clermont-Ferrand, France; acomptour@chu-clermontferrand.fr (A.C.); pchauvet@chu-clermontferrand.fr (P.C.); cfiguier@chu-clermontferrand.fr (C.F.); asgremeau@chu-clermontferrand.fr (A.-S.G.); mcanis@chu-clermontferrand.fr (M.C.); 2 INSERM, CIC 1405, Unité CRECHE, 63000 Clermont-Ferrand, France; 3 CHU Clermont-Ferrand, Unité de Biostatistiques (DRCI), 63000 Clermont-Ferrand, France; clambert@chu-clermontferrand.fr (C.L.); bpereira@chu-clermontferrand.fr (B.P.); 4 UMR6602, Endoscopy and Computer Vision Group, Faculté de Médecine, Institut Pascal, Bâtiment 3C, 28 place Henri Dunant, 63000 Clermont-Ferrand, France

**Keywords:** endometriosis, quality of life, pain, group-based trajectory modeling

## Abstract

Many studies have shown a global efficacy of laparoscopic surgery for patients with endometriosis in reducing painful symptoms and improving quality of life (QoL) in the short and long-term. The aim of this study was to analyze the different trajectories of long-term evolution in QoL and symptoms following surgical treatment for endometriosis, and to identify corresponding patient profiles. This prospective and multicenter cohort study concerned 962 patients who underwent laparoscopic treatment for endometriosis. QoL was evaluated using the Short Form (SF)-36 questionnaire and intensity of pain was reported using a visual analog scale prior to surgery and at 6, 12, 18, 24 and 36 months after surgery. Distinctive trajectories of pain and QoL evolution were identified using group-based trajectory modeling, an approach which gathers individuals into meaningful subgroups with statistically similar trajectories. Pelvic symptom trajectories (models of the evolution of dysmenorrhea, dyspareunia and chronic pelvic pain intensity over years) correspond to (1) patients with no pain or pain no longer after surgery, (2) patients with the biggest improvement in pain and (3) patients with continued severe pain after surgery. Our study reveals clear trajectories for the progression of symptoms and QoL after surgery that correspond to clusters of patients. This information may serve to complete information obtained from epidemiological methods currently used in selecting patients eligible for surgery.

## 1. Introduction

Many studies have shown the efficacy of laparoscopic surgery for patients with endometriosis in reducing painful symptoms and improving quality of life (QoL) in the short term [1,2,3,4,5,6] and some of them in the long-term [7,8,9,10,11,12,13,14,15,16]. We previously analyzed the evolution of pain (dysmenorrhea, chronic pelvic pain, dyspareunia) and quality of life in this cohort over 3 years of follow-up, specifically focusing on the impact of surgical treatment of endometriosis [8]. We reported a global improvement in all reported symptoms and all QoL scores (physical and mental components) as early as six months after surgery. After one year, QoL and symptom improvements remained unchanged over a long-term period [8]. In addition to studying the evolutionary kinetics of QoL and pain, the aim of this study was to (i) analyze long-term individual trajectories following surgical treatment for endometriosis using group-based trajectory modeling and (ii) identify which patient types are related to these trajectories [17]. This trajectory approach allows for an original study identifying groups of patients according to the evolution of pelvic pain intensity (using a visual analog scale (VAS) for dyspareunia, dysmenorrhea and chronic pelvic pain) and of Physical Component Summary (PCS) and Mental Component Summary (MCS) scores (using the Short Form (SF)-36 questionnaire).

## 2. Methods

### 2.1. Study Approval

Institutional Review Board approval was obtained in 2003 (PHRC 2003 R.05-04).

### 2.2. Patients and Methods

This prospective multicenter study was previously presented [8,18] and concerned a cohort of 962 consecutive patients aged 15–50 years treated by laparoscopy between January 2004 and December 2012.

Having obtained informed written consent, data on epidemiology and medical history were collected prospectively at the time of inclusion using a case report form. Surgeons completed operative reports, precisely detailing diagnostic lesions and grading in accordance with revised American Fertility Society (rAFS) scores, and treatment given. Symptoms (painful gynecological or digestive symptoms), quality of life and fertility self-report written questionnaires were collected at different time points: prior to surgery (t0), 6, 12, 18, 24 and 36 months after surgery.

Patients were either recruited when scheduling surgery or the day before surgery. Of the 1237 patients operated on for suspected endometriosis between January 2004 and December 2012, 962 patients were enrolled, 92 patients had no histological confirmation of endometriosis, 25 did not fulfill inclusion criteria, 139 refused participation in the study and 19 had missing data for the trajectory analysis. The response rate was 79% at 12 months after surgery [8]. Patients lost to follow-up included those who declined further participation in the study and those who could not be contacted due to a change in address or telephone number. Intensity of pain was reported using a visual analog scale (VAS) (0 represents no pain and 10 the worst imaginable pain), and quality of life was evaluated using the French version of the Medical Outcome Study Short Form 36 Item Health Survey (MOS SF-36) [8,18,19,20]. Pelvic pain symptoms (dysmenorrhea, chronic pelvic pain (Cpp) and dyspareunia) have been previously described [18,21]. Medical treatment prescribed after surgery included contraception (oral progestins, the Mirena intrauterine device, implant, continuous pills), Luteinizing Hormone Releasing Hormone (LHRH) analogues or antigonadotropic agents.

### 2.3. Surgical Technique

Our surgical technique has been previously described [18]. Laparoscopy being the preferred approach, surgical treatment of endometriosis was performed as follows: superficial peritoneal lesions were treated by bipolar coagulation or peritoneal resection; endometriomas were treated by cystectomy as described previously [22]; recto-vaginal nodules were treated by shaving or by colorectal resection where a satisfactory shaving procedure was deemed impossible (our surgical technique has been previously reported [11,23]). Shaving was performed using the “reverse technique” [8,23] starting by the lateral and the vaginal parts of the nodule and ending with rectal invasion. The nodule was dissected all around the rectal involvement so as to provide essential exposure of the rectal part of the nodule for the most difficult part of the procedure [23]. The need for colorectal resection was reevaluated after shaving was completed and primarily involved consideration of the following factors: residual disease, persistence of bowel stenosis (lesions involving more than 130 to 150 of the rectum), opening of the digestive lumen without the possibility of satisfactory suture (tension-free suture using healthy surrounding tissue), and patient digestive preoperative symptoms. The final decision was made with the gastro-intestinal surgeon and took into consideration vaginal opening, adhesions related to previous surgeries, technical difficulties and co-morbidities [23]. In cases of endometriosis invading the sigmoid colon or the small bowel, segmental resection was performed. Ureteral involvement was treated by ureteroneocystostomy when necessary as previously described [24]. In cases of bladder lesion invading the mucosa, segmental resection was performed and closed using single or double layer suturing. Laparoscopic techniques, operation devices, and standards of postoperative care remained unchanged during the entire period of the cohort.

### 2.4. Statistical Analyses

All analyses were performed using Stata software (Version 13, StataCorp, College Station, TX, USA) for a two-sided Type I error of 5%. Patient characteristics were expressed as mean ± standard-deviation or median and interquartile range, for continuous data (assumption of normality assessed by using the Shapiro—Wilk test) and as numbers and associated percentages for categorical parameters.

To identify distinctive trajectories for evolution of pain and QoL, a group-based trajectory model (GBTM) was performed. GBTM involves an approach that gathers individuals into meaningful subgroups that show statistically similar trajectories, i.e., identify groups based on distinctive trajectories [25,26]. This analysis provides a formal way to determine the best-fit number of trajectories and a precision estimate of group membership allocation which can be expressed using observed probabilities and posteriori probabilities, with values expected to be close. The posterior probabilities of group membership measures the likelihood of each patient to belong to an assigned group. Nagin et al. recommends that average posterior probabilities should exceed a minimum of 0.70 for each group [27].

Continuous variables were compared between independent trajectory groups by ANOVA, or Kruskal—Wallis test when assumptions for ANOVA were not met. Homoscedasticity was analyzed using the Bartlett’s test. When appropriate, post-hoc tests were performed taking into account multiple comparisons (Tukey–Kramer post ANOVA and Dunn’s test after Kruskal—Wallis). Comparisons between independent trajectories were carried out using Chi-squared or Fisher’s exact tests for categorical variables. When appropriate, a post-hoc test was performed (Marascuillo procedure). Relationships between pain, quality-of-life trajectories and outcomes were analyzed as mentioned above.

These tests were completed by multiple correspondence analysis (MCA) and a mixed unsupervised classification (k-means clustering applied to the partition obtained from an ascending hierarchical classification using Ward’s distance) in order to determine patient profiles (clusters of individuals sharing closely similar characteristics) categorized according to relationships between modalities of the variables. The parameters included in the MCA were selected by clinical relevance: demographic data, gynecological and digestive symptoms, psychological disorders, QoL, fertility, rAFS Stage and preoperative medical treatment. Finally, the association between trajectories (determined by GBTM) and patient profiles (determined by MCA) was studied.

Performing the MCA necessitates a complete data set, i.e., no missing data for any variable used in the analysis. A sensitivity analysis was carried out to evaluate representativeness and compared the MCA dataset with that of all the patients. As recommended by several statisticians [28], particular focus was given to the magnitude of differences. For the GBTM approach, each patient was required to have at least two measures at two different times. This assumption being met, all patients were included in the analysis.

## 3. Results

### 3.1. Description of the Population

Characteristics of the population are detailed in Table 1 and previously in the literature [8,18].

### 3.2. Trajectories

**Figure 1:**
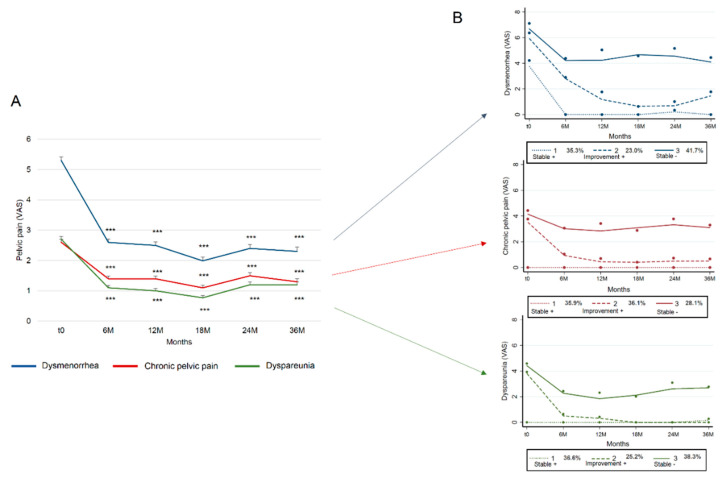
Trajectories of the long-term evolution of pelvic symptoms following surgical treatment for endometriosis. (**A**) Evolution of pelvic pain following surgical treatment; (**B**) Trajectories for each pelvic pain. For graph A, each curve represents mean ± standard error of the mean; ***: *p* < 0.001 compared to baseline. VAS: visual analog scale.

For all patients, the mean VAS score for dysmenorrhea fell from 5.3 ± 3.7 at baseline to 2.6 ± 3.3 at 6M, 1.99 ± 3.2 at 18M and 2.3 ± 3.3 at 36M of follow-up (*p* < 0.001) (Figure 1A). Mean VAS scores for Chronic pelvic pain (Cpp)nd dyspareunia fell from 2.6 ± 3.5 and 2.7 ± 3.2 respectively prior to surgery to 1.4 ± 2.5 and 1.1 ± 2.2 at 6M, 1.4 ± 2.5 and 1.0 ± 2.2 at 12M, then 1.3 ± 2.5 and 1.2 ± 2.3 at 36M of follow-up. For all VAS scores analyzed, no significant difference was observed between postoperative time points. After applying the GBTM approach for each pelvic symptom, trajectory 1 (Stable +) corresponded to patients who reported no pain before surgery (or a VAS pain score of 4 for dymenorrhea) and after surgery over a long-term period (Figure 1B). Trajectory 2 (Improvement +) corresponded to patients with preoperative pain (VAS 4–6 depending on symptom type) and a large decrease in VAS score long term after surgery. Trajectory 3 (Stable −) corresponded to patients with severe preoperative pain (VAS 4–7 depending on symptom type) with long term reduced but persistent pain levels after surgery, (VAS 2–4.5 depending on symptom type).

For dysmenorrhea, 35.3% of patients were in trajectory 1 (Stable +), 23.0% in trajectory 2 (Improvement +) and 41.7% in trajectory 3 (Stable −) (observed probabilities), with posterior probabilities of 29.9%, 32.2%, 38.0%, average posterior probabilities of 79.0%, 83.4% and 78.9% (expected >70%) and odds of correct classification based on posterior probabilities of group membership of 6.9, 16.9 and 5.2 (expected >5) (Figure 1B, Table 2) [29]. For Cpp, 35.9% of patients were in trajectory 1 (Stable +), 36.1% in trajectory 2 (Improvement +) and 28.1% in trajectory 3 (Stable −) (observed probabilities), with posterior probabilities of 28.1%, 40.4%, 31.5%, average posterior probabilities of 78.4%, 82.2% and 84.5% and odds of correct classification based on posterior probabilities of group membership of 6.5, 8.2 and 14 (Figure 1B, Table 2). For dyspareunia, 36.6% of patients were in trajectory 1 (Stable +), 25.2% in trajectory 2 (Improvement +) and 38.3% in trajectory 3 (Stable −), with posterior probabilities of 29.9%, 30.4%, 39.7%, average posterior probabilities of 81.7%, 84.2% and 90.0% and odds of correct classification based on the posterior probabilities of group membership of 7.7, 15.9 and 14.5 (Figure 1B, Table 2).

**Figure 2:**
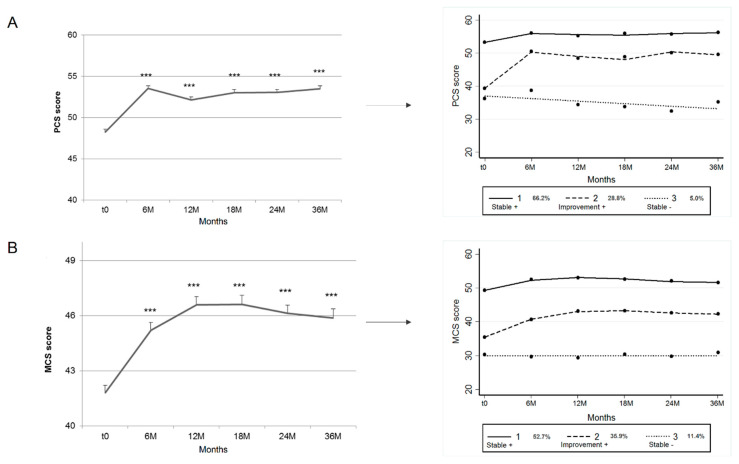
Trajectories of the long-term evolution of quality of life (PCS and MCS) following surgical treatment for endometriosis. For graphs (**A**) (PCS) and (**B**) (MCS), each curve represents mean ± standard error of the mean; ***: *p* < 0.001 compared to baseline. MCS: mental component summary; PCS: physical component summary.

For PCS and MCS (Figure 2), trajectory 1 (Stable +) concerned patients with the highest QoL which remained stable before and after surgery (scores 52–58 for PCS and 49–53 for MCS). Trajectory 2 (Improvement +) concerned patients with altered preoperative QoL (scores 38 for PCS and 35 for MCS) with increased QoL after surgery (scores 50 for PCS and 42–43 for MCS) that remained stable over a period of years. Trajectory 3 (Stable −) concerned patients with severe altered preoperative QoL (approximate score 32–38 for PCS and 30 for MCS), which remained stable over a period of years. For PCS, 66.2% of patients were in trajectory 1, 28.8% in trajectory 2 and 5.0% in trajectory 3 (observed probabilities), with posterior probabilities of 65.2.%, 28.9%, 6.0%, average posterior probabilities of 92%, 80.6% and 88.1% and odds of correct classification based on the posterior probabilities of group membership equal to 5.9, 10.3 and 140.8 (Figure 2A, Table 2). For MCS, 52.7% of patients were in trajectory 1, 35.9% in trajectory 2 and 11.4% in trajectory 3 (observed probabilities), with posterior probabilities of 51.3%, 35.0%, 13.7%, average posterior probabilities of 89.1%, 76.6% and 82.4% and odds of correct classification based on the posterior probabilities of group membership equal to 7.3, 5.9 and 36.4 (Figure 2B, Table 2).

### 3.3. Comparison of Pelvic Pain and Quality-of-Life Trajectories

Pelvic symptom trajectories correspond to (1) patients with no pain or pain no longer after surgery (Stable +), (2) patients with the biggest improvement in pain (Improvement +), (3) patients with continued severe pain after surgery (Stable −).

PCS and MCS trajectories correspond to: (1) patients with the highest QoL score which remained stable over a period of years (Stable +); (2) patients with the biggest improvement in QoL score (Improvement +); and (3) patients with a poor QoL score maintained after surgery (Stable −).

#### 3.3.1. PCS and Pelvic Symptoms

**Figure 3:**
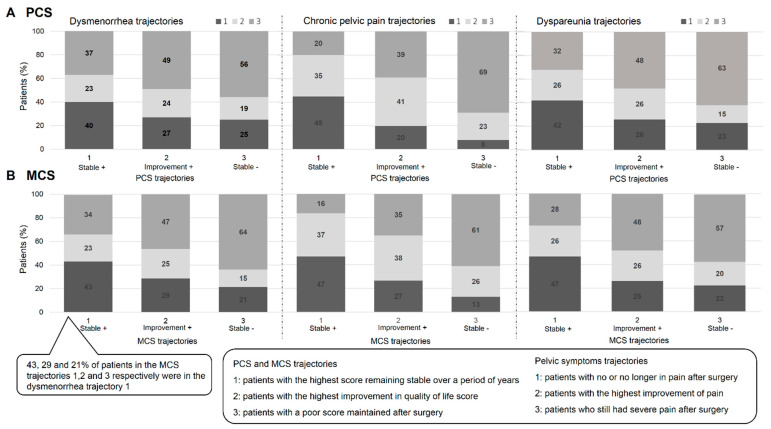
Comparison of pelvic pain and quality-of-life trajectories. (**A**) PCS score; (**B**) MCS score. MCS: mental component summary; PCS: physical component summary.

Dysmenorrhea: 40% of patients in the PCS Stable + trajectory were also in the dysmenorrhea Stable + trajectory; 24% of patients in the PCS Improvement + trajectory were also in the dysmenorrhea Improvement + trajectory; 56% of patients in the PCS Stable − trajectory were also in the dysmenorrhea Stable – trajectory (Figure 3A).

Cpp: 45%, 41% and 69% of patients in the PCS Stable +, Improvement + and Stable − trajectories respectively were also in the Cpp Stable +, Improvement + and Stable − Cpp trajectories respectively (Figure 3A).

Dyspareunia: 42%, 26% and 63% of patients in the PCS Stable +, Improvement + and Stable – trajectories respectively were also in the dyspareunia Stable +, Improvement + and Stable − trajectories respectively (Figure 3A).

#### 3.3.2. MCS and Pelvic Symptoms

Dysmenorrhea: 43%, 25% and 64% of patients in the MCS Stable +, Improvement + and Stable − trajectories respectively were in the dysmenorrhea Stable +, Improvement + and Stable − trajectories respectively (Figure 3B).

Cpp: 47%, 38% and 61% of patients in the MCS Stable +, Improvement + and Stable − trajectories respectively were also in the dysmenorrhea Stable +, Improvement + and Stable − trajectories respectively (Figure 3B).

Dyspareunia: 47%, 26% and 57% of patients in the MCS Stable +, Improvement + and Stable − trajectories respectively were in the dysmenorrhea Stable +, Improvement + and Stable − trajectories respectively (Figure 3B).

### 3.4. Multiple Correspondence Analysis (MCA) (at t0)

To determine clusters of individuals sharing closely similar characteristics, categorized according to relationships between modalities of the variables, the aforementioned univariate analyses were completed using the MCA approach (Supplemental Figure S1). The clusters are described in Table 3 according to the variables of interest (not all variables used are shown). Cluster 1 (*n* = 76) included only 20% of patients under 29 years old, 45% with infertility antecedents, 37% for whom pain was the main reason for intervention, 22%, 1% and 6% for whom VAS scores for pelvic pain (dysmenorrhea, Cpp and dyspareunia respectively) were superior or equal to 7; 17% and 24% of patients reported a PCS score <50 and a MCS score <40 respectively. Cluster 2 (*n* = 182) included 36% of patients under 29 years old, 57% with infertility antecedents and 65% with pregnancy desire, 52% for whom pain was the main reason for intervention, 86%, 4% and 15% for whom VAS scores for pelvic pain (dysmenorrhea, Cpp and dyspareunia respectively) were superior or equal to 7; 42% and 37% reported a PCS score <50 and a MCS score <40 respectively. Cluster 3 (*n* = 102) included 35% of patients under 29 years old, 45% with infertility antecedents and 52% with pregnancy desire, 4% with a family history of endometriosis, 60% for whom pain was the main reason for intervention, 42% who had preoperative medical treatment, 39% reported digestive symptoms (dyschezia or constipation), 18% reported painful urination, 77%, 32% and 33% for whom VAS scores for pelvic pain (dysmenorrhea, Cpp and dyspareunia respectively) were superior or equal to 7; 67% and 53% reported a PCS score <50 and a MCS score <40 respectively. Cluster 4 (*n* = 62) included 42% of patients under 29 years old, 29% with infertility antecedent and 52% with pregnancy desire, 18% with a family history of endometriosis, 76% for whom pain was the main reason for intervention, 45% who had preoperative medical treatment, 48% and 37% reported digestive symptoms (dyschezia and constipation respectively), 15% reported painful urination, 87%, 73% and 32% for whom VAS scores for pelvic pain (dysmenorrhea, Cpp and dyspareunia respectively) were superior or equal to 7; 84% and 69% reported a PCS score <50 and a MCS score <40 respectively. rAFS was not significantly different between the 4 clusters (Table 3).

### 3.5. Comparison of Clusters and Trajectories

**Figure 4:**
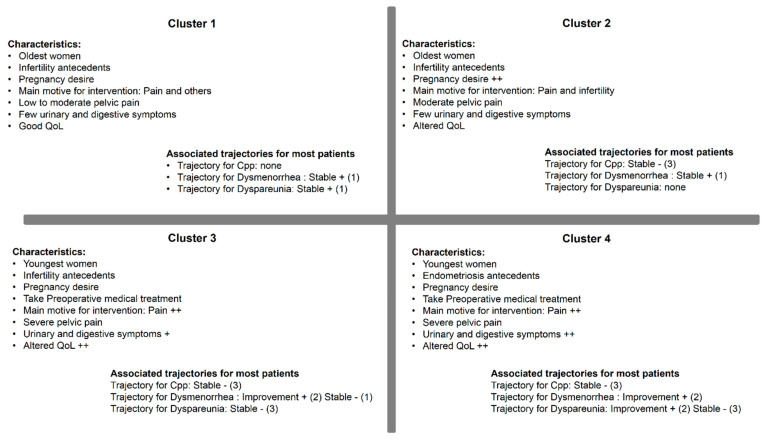
Characteristics of each cluster of patients. Cpp: chronic pelvic pain; QoL: quality of life.

More than 50% of patients from Clusters 1 and 2 and none from Clusters 3 and 4 were in the dysmenorrhea trajectory 1 (Stable +) (Table 4, Figure 4). By contrast, more than 50% of patients from Cluster 3 and Cluster 4 were in the dysmenorrhea trajectory 2 (Improvement +) (*p* < 0.001).

Most patients from Clusters 2, 3 and 4 (49%, 51% and 48% respectively) were in the Cpp trajectory 3 (Stable −) though this result was not statistically significant (*p* = 0.16).

A majority of patients from Cluster 1 were in dyspareunia trajectory 1 (Stable +) and most patients from Clusters 2, 3 and 4 were in the dyspareunia trajectory 3 (Stable −) (*p* = 0.001).

### 3.6. Profiles of Patients from Different Trajectories: Univariate Analyses

Patient profiles from trajectory 3 (Stable −) for each pelvic symptom and including PCS and MCS scores revealed patients to be younger (most were single), the majority were smokers, patients with severe symptoms and the highest VAS scores for pelvic, digestive and urinary symptoms. These patients also took the most analgesics, reported the most mental disorders (fatigue, headache, anxiety, depression, sleep disorders) and had the most altered physical and mental QoL (Tables S1 and S2). Patients from trajectory 1 (Stable +) for each pelvic symptom and including PCS and MCS scores were older patients, who reported the least severe pain and a good quality of life.

## 4. Discussion

This study highlights the heterogeneity of long-term symptoms and quality of life experienced by patients having undergone a first surgery for endometriosis. For pelvic pain, the group-based trajectory modeling method revealed three categories of patients: 25–36% of patients experienced improvement in pelvic pain six months after surgery, 28–41% of patients continued to report moderate pelvic pain after surgery over a period of years. The remaining patients (approximately 35–37%) with little or no pain prior to surgery, had no postoperative pain over a period of years. Overall, each trajectory corresponded to 1/3 of patients: 1/3 with considerable improvement, 1/3 with moderate improvement but persistent symptoms and 1/3 of stable patients with little or no remaining symptoms.

For QoL, the group-based trajectory modeling method also revealed three categories of patients: a few patients (5–11%) for whom poor QoL remained unchanged after surgery, 28–35% of patients who after surgery reported the greatest improvement in quality-of-life scores and a majority of patients (53–67%) with the highest QoL scores before surgery remaining stable over a period of years. The benefits of surgery are mostly experienced by patients in trajectory 2 for pelvic pain and QoL. Our analysis included only PCS and MCS; it would also be interesting to include all dimensions of PCS (physical functioning (PF), role limitations—physical (RP) and bodily pain (BP) and MCS (vitality (VT), social functioning (SF), role limitations—emotional (RE), mental health (MH) and general health (GH)).

The MCA method revealed four Clusters of patients before surgery. The majority of patients in Clusters 1 and 2 were older and presented with infertility antecedents and pregnancy desire. They reported few low-intensity symptoms (notably Cluster 1) and good QoL. Most patients in Cluster 3 and Cluster 4 were younger, had a pregnancy desire and had fewer infertility antecedents (notably Cluster 4); they reported the most severe symptoms (notably Cluster 4), a greater use of antalgics and an altered QoL. A comparison between clusters and trajectories revealed similar tendencies: a majority of patients from Cluster 1 and Cluster 2 who had little or no pelvic pain prior to surgery no longer experienced pain after surgery. The remaining small number of patients had persistent pain after surgery. Among patients from Cluster 3 and Cluster 4, a majority of those who reported severe dysmenorrhea prior to surgery experienced relief from symptoms after, however a majority of those who reported Cpp and dyspareunia continued to experience intense/moderate pelvic pain after surgery.

Although surgery is effective for most patients, some patient profiles (notably in trajectory 2) appear to be more eligible for surgery than others as they experience greater benefits [18,30]. In a previous article, we highlighted that pelvic pain was the most significant independent predictive factor for improvement in QoL after surgery. Patients with pelvic pain appear to benefit the most from surgery in terms of QoL.

We also previously suggested that differing patient evolutions in pain and QoL after surgery may be explained by postoperative side effects, complications, disease recurrences and persistent and recurrent pain after surgery [18]. Recurrence of endometriosis is not uncommon and recurrence of pain is frequent, though the two may not be associated [11,31,32]. In addition, even if pain severity does not necessarily correlate with the extent of the disease [33], pain localization tends to correlate with where lesions are situated [33].

This study revealed a further finding of interest: although a correlation exists between improvement in pain and QoL (see Figure 3), the improvement of the former does not only depend on that of the latter or vice versa. Some patients reported a high QoL score that was maintained after surgery, while also reporting continued severe pain. Other patients reported poor QoL despite no longer suffering from pain (Figure 3) [34].

Pain is multifactorial and painful symptoms do not only arise due to endometriotic lesions. Key to patients benefiting from the long term positive effects of laparoscopic surgery is a multidisciplinary pre assessment and a post-operative follow-up (pre-operative imaging by a radiologist, medical and surgical management by a multidisplinary surgical team should be associated with complementary supportive care treatments (psychological therapy, physiotherapy) and alternative medicines (osteopathy, acupuncture, etc.)) [18,35,36,37].

The main limitation of our study relates to missing data. GBTM analysis was based on the assumption that each patient required at least two time-points for evaluation, but due to missing data the same time-points were not possible for all patients. A sensitivity analysis carried out using a last observation carried forward (LOCF) missing data approach, however, confirmed our results. For example, LOCF applied to dysmenorrhea similarly highlighted three trajectories, revealing the following posterior probabilities: 31.3%, 32.7% and 36% (vs. 29.8%, 32.2%, 38.0% without LOCF) with more than 75% of patients classified in the same trajectories. Secondly, due to missing data, MCA was not carried out with the same sample as for GBTM (*n* = 422 vs. *n* = 962 respectively). Representativeness analysis however confirmed that the two samples were comparable. Despite missing data, sensitivity analyses provided strong support for our conclusions. Furthermore, although the SF-36 questionnaire has been validated for use with endometriosis and has been largely used in studies [34], it remains a generic scale and fails to address specific domains relating to the disease, such as sexual functioning. The EHP-30 scale would provide a more adapted method for studying the impact of endometriosis on patient health-related quality of life. But when this cohort was designed, the EHP-30 questionnaire was not yet used or validated in French [38,39]. Thirdly, taking into account post-operative medical treatment into the analysis of QoL trajectories was very difficult because follow-up of medical therapies was complicated to undertake, with statistical analysis hampered due to missing data or small sample size per subgroup of treatment [18].

## 5. Conclusions

Certain patient profiles are more likely to undergo surgery than others, however it remains difficult to accurately predict which patients will benefit from improvement in pain and quality of life. We have revealed, for the first time, clear trajectories for the progression of symptoms and quality of life after surgery that correspond to clusters of patients. This information may serve to complete that obtained from epidemiological methods currently used in selecting patients eligible for surgery. Future research is required to develop mathematical models or the use of artificial intelligence, so as to both exploit the data from these trajectories and to allow patients to be associated with a trajectory at the time of preoperative consultation.

## Figures and Tables

**Table 1:** Characteristics of the population.

	*n* = 962	*n* = 540	*n* = 422
**Demographic Data**			
Age (years)	33.0 ± 7.1	34.0 ± 7.5	31.8 ± 6.3
Tobacco (%)	36.7	36.9	36.5
BMI (kg/m^2^)	22.1 ± 3.7	22.2 ± 4.0	22.0 ± 3.4
In couple (%)	78.0	76.0	80.6
**Antecedent**			
Endometriosis antecedents (%)	10.3	12.0	8.3
Preoperative medical treatment (%)	29.2	27.5	31.3
Infertility (%)	41.7	36.8	47.9
**Symptoms**			
Dysmenorrhea (VAS)	6.0 ± 3.4	5.0 ± 3.9	7.2 ± 2.1
Chronic pelvic pain (VAS)	2.9 ± 3.4	2.8 ± 3.5	3.1 ± 3.4
Dyspareunia (VAS)	3.0 ± 3.2	2.7 ± 3.2	3.4 ± 3.2
**Preoperative Characteristics**			
Pregnancy desire (%)	53.1	49.0	57.8
Period between first consultation and diagnosis (years)	6 (2; 15)	5 (2; 15)	6 (2; 15)
Pelvic ultrasound (%)	94.5	93.9	95.3
MRI (%)	23.9	23.4	24.5
Motive for intervention (%)			
Pain	51.4	49.0	54.5
Sterility	20.6	18.0	23.9
Endometriosis	13.7	16.1	10.7
Others	14.3	16.9	10.9
**Operative Characteristics**			
rAFS stage (%)			
Stage I: minimal	27.4	26.0	29.1
Stage II: mild	28.3	29.6	26.8
Stage III: moderate	20.7	20.3	21.1
Stage IV: severe	23.6	24.1	23.0
Duration of surgery (minutes)	75 (60; 120)	75 (60; 120)	80 (55; 120)
**Procedure**			
Surgical procedure (%)	96.5	96.2	96.9
Adhesiolysis (%)	61.0	61.5	60.5
Peritoneal superficial lesions (%)	73.9	69.6	79.3
Ovarian endometrioma (%)	47.8	50.0	45.0
Endometriosis nodule (%)	58.0	58.0	57.9
Digestive intervention (%)	4.9	5.6	4.1
Urological intervention (%)	7.1	7.4	6.7
Postoperative medical treatment (%)	44.2	40.4	49.2

Characteristics of the population (*n* = 962). Comparison with patients included in the multiple correspondence analysis (*n* = 422) and other patients (those not included in the multiple correspondence analysis (*n* = 540)). Data are presented as percentages, mean ± standard deviation or median (interquartile range). BMI: Body Mass Index; MRI: magnetic resonance imaging; rAFS: revised American fertility society; VAS: visual analog scale.

**Table 2:** Characteristics of pelvic pain and quality-of-life trajectories.

Dysmenorrhea			
	Trajectory 1 (Stable +)	Trajectory 2 (improvement +)	Trajectory 3 (Stable −)
Observed probabilities	35.3	23.0	41.7
Posterior probabilities	29.9	32.2	38.0
Average posterior probabilities	79.0	83.4	78.9
Odds of correct classification	6.9	16.9	5.2
**Cpp**			
	Trajectory 1 (Stable +)	Trajectory 2 (improvement +)	Trajectory 3 (Stable −)
Observed probabilities	35.9	36.1	28.1
Posterior probabilities	28.1	40.4	31.5
Average posterior probabilities	78.4	82.2	84.5
Odds of correct classification	6.5	8.2	14
**Dyspareunia**			
	Trajectory 1 (Stable +)	Trajectory 2 (improvement +)	Trajectory 3 (Stable −)
Observed probabilities	36.6	25.2	38.3
Posterior probabilities	29.9	30.4	39.7
Average posterior probabilities	81.7	84.2	90.0
Odds of correct classification	7.7	15.9	14.5
**PCS**			
	Trajectory 1 (Stable +)	Trajectory 2 (improvement +)	Trajectory 3 (Stable −)
Observed probabilities	66.2	28.8	5.0
Posterior probabilities	65.2	28.9	6.0
Average posterior probabilities	92	80.6	88.1
Odds of correct classification	5.9	10.3	140.8
**MCS**			
	Trajectory 1 (Stable +)	Trajectory 2 (improvement +)	Trajectory 3 (Stable −)
Observed probabilities	52.7	35.9	11.4
Posterior probabilities	51.3	35.0	13.7
Average posterior probabilities	89.1	76.6	82.4
Odds of correct classification	7.3	5.9	36.4

Data are presented as percentages. Cpp: chronic pelvic pain

**Table 3:** Characteristics of the four clusters obtained by multiple correspondence analysis. Data are presented as percentages.

	Cluster 1 (*n* = 76)	Cluster 2 (*n* = 182)	Cluster 3 (*n* = 102)	Cluster 4 (*n* = 62)	*p*-Value
Age (years)					0.05
<29	19.7	35.7	35.3	41.9	
29 to 34	47.4	40.1	33.3	27.4	
≥35	32.9	24.2	31.4	30.7	
Body mass index < 25 kg/m^2^	86.8	86.8	82.4	83.9	0.73
Tobacco	30.3	33	44.1	41.9	0.13
Single	26.3	16.5	20.6	17.7	0.32
Menarche ≤ 12 years	40.8	48.9	49	43.5	0.6
Infertility antecedents	44.7	57.1	45.1	29	0.001
Endometriosis antecedents	6.6	8.2	3.9	17.7	0.02
Pregnancy desire	53.9	64.8	52	51.6	0.09
Preoperative medical treatment	23.7	23.6	42.2	45.2	<0.001
Main motive for intervention					<0.001
Pain	36.8	51.6	59.8	75.8	
Sterility	23.7	31.9	16.7	12.9	
Endometriosis	11.8	8.8	16.7	4.8	
Other	27.6	7.7	6.9	6.5	
VAS dysmenorrhea					<0.001
<3	17.1	0	2	0	
3 to 6	60.5	14.3	20.6	12.9	
≥7	22.4	85.7	77.4	87.1	
VAS chronic pelvic pain					<0.001
<3	82.9	80.8	8.8	1.6	
3 to 6	15.8	15.4	58.8	25.8	
≥7	1.3	3.8	32.4	72.6	
VAS dyspareunia					<0.001
<3	65.8	44.5	33.3	30.6	
3 to 6	27.6	40.1	33.3	37.1	
≥7	6.6	15.4	33.3	32.3	
Dyschezia	14.5	39.6	39.2	48.4	<0.001
Nausea	5.3	28.6	39.2	40.3	<0.001
Rectal bleeding	2.6	3.8	2.9	11.3	0.08
Constipation	15.8	30.2	39.2	37.1	0.006
Diarrhea	17.1	28.6	26.5	33.9	0.14
Painful urination	3.9	10.4	17.6	14.5	0.03
Fatigue	40.8	59.3	76.5	83.9	<0.001
Headache	26.3	42.9	52.9	56.5	0.001
PCS ≤ 50	17.1	42.3	66.7	83.9	<0.001
MCS ≤ 40	23.7	36.8	52.9	69.4	<0.001
Analgesic treatment during menstruations	59.2	92.3	86.3	91.9	<0.001
Analgesic treatment outside menstruations	6.6	6	50	77.4	<0.001
rAFS stage					0.48
Stage I: minimal	38.2	25.8	32.4	22.6	
Stage II: mild	21.1	28.6	28.4	25.8	
Stage III: moderate	21.1	19.8	21.6	24.2	
Stage IV: severe	19.7	25.8	17.6	27.4	

rAFS: revised American Fertility Society.

**Table 4:** Crossing of the different trajectories with Clusters. Cpp: chronic pelvic pain.

	Clusters				
	1	2	3	4	*p*-Value
	(*n* = 76)	(*n* = 182)	(*n* = 102)	(*n*= 62)	
Trajectories of Cpp					0.16
1	32.9	28.0	26.5	24.2	
2	35.5	23.1	22.5	27.4	
3	31.6	48.9	51.0	48.4	
Trajectories of dysmenorrhea					<0.001
1	52.6	51.7	0.0	0.0	
2	35.5	28.0	53.9	59.7	
3	11.8	20.3	46.1	40.3	
Trajectories of dyspareunia					0.001
1	47.4	32.4	18.6	19.4	
2	25.0	29.7	29.4	37.1	
3	27.6	37.9	52.0	43.5	

## Supplementary Materials

The following are available online at https://www.mdpi.com/2077-0383/9/8/2461/s1, Figure S1: Variable categories (A) and individual positioning (B) on the two first axes following multiple correspondence analysis (MCA). (A) Relationships between variable categories can be interpreted as follows: categories with a similar profile are close to each other, negatively correlated categories are plotted on opposite sides of the origin, the distance between categories and the origin indicates category quality (distant points from the origin are well represented by the MCA). (B) Factorial analysis revealed 4 patient profiles prior to surgery. Table S1: Characteristics of patients from different pelvic pain trajectories: univariate analyses, Table S2: Characteristics of patients from different MCS and PCS trajectories: univariate analyses.

## References

[1] Mabrouk M., Montanari G., Guerrini M., Villa G., Solfrini S., Vicenzi C., Mignemi G., Zannoni L., Frasca C., Di Donato N. (2011). Does laparoscopic management of deep infiltrating endometriosis improve quality of life? A prospective study. Heal. Qual. Life Outcomes.

[2] Vercellini P., Aimi G., Busacca M., Apolone G., Uglietti A., Crosignani P.G. (2003). Laparoscopic uterosacral ligament resection for dysmenorrhea associated with endometriosis: Results of a randomized, controlled trial. Fertil. Steril..

[3] Ribeiro P.A., Sekula V.G., Abdalla-Ribeiro H.S., Rodrigues F.C., Aoki T., Aldrighi J.M. (2013). Impact of laparoscopic colorectal segment resection on quality of life in women with deep endometriosis: One year follow-up. Qual. Life Res..

[4] Bassi M.A., Podgaec S., Dias J.A., Filho N.D., Petta C.A., Abrão M.S. (2011). Quality of Life after Segmental Resection of the Rectosigmoid by Laparoscopy in Patients with Deep Infiltrating Endometriosis with Bowel Involvement. J. Minim. Invasive Gynecol..

[5] Parra R.S., Feitosa M.R., De Camargo H.P., Valério F.P., Zanardi J.V.C., Da Rocha J.J.R., Féres O. (2020). The impact of laparoscopic surgery on the symptoms and wellbeing of patients with deep infiltrating endometriosis and bowel involvement. J. Psychosom. Obstet. Gynecol..

[6] Riiskjær M., Forman A., Kesmodel U.S., Andersen L.M., Ljungmann K., Seyer-Hansen M. (2018). Pelvic Pain and Quality of Life Before and After Laparoscopic Bowel Resection for Rectosigmoid Endometriosis. Dis. Colon Rectum.

[7] Abbott J., Hawe J., Clayton R., Garry R. (2003). The effects and effectiveness of laparoscopic excision of endometriosis: A prospective study with 2–5 year follow-up. Hum. Reprod..

[8] Comptour A., Chauvet P., Canis M., Gremeau A.-S., Pouly J.-L., Rabischong B., Pereira B., Bourdel N. (2019). Patient Quality of Life and Symptoms after Surgical Treatment for Endometriosis. J. Minim. Invasive Gynecol..

[9] Byrne D., Curnow T., Smith P., Cutner A., Saridogan E., Clark T.J., Saraswat L. (2018). Laparoscopic excision of deep rectovaginal endometriosis in BSGE endometriosis centres: A multicentre prospective cohort study. BMJ Open.

[10] Darai E., Dubernard G., Coutant C., Frey C., Rouzier R., Ballester M. (2010). Randomized Trial of Laparoscopically Assisted Versus Open Colorectal Resection for Endometriosis. Ann. Surg..

[11] Bourdel N., Comptour A., Bouchet P., Gremeau A.-S., Pouly J.-L., Slim K., Pereira B., Canis M. (2017). Long-term evaluation of painful symptoms and fertility after surgery for large rectovaginal endometriosis nodule: A retrospective study. Acta Obstet. Gynecol. Scand..

[12] Arcoverde F.V.L., Andres M.P., Borrelli G.M., Barbosa P.D.A., Abrão M.S., Kho R.M. (2019). Surgery for Endometriosis Improves Major Domains of Quality of Life: A Systematic Review and Meta-Analysis. J. Minim. Invasive Gynecol..

[13] Turco L.C., Scaldaferri F., Chiantera V., Cianci S., Ercoli A., Fagotti A., Fanfani F., Ferrandina G., Nicolotti N., Tamburrano A. (2019). Long-term evaluation of quality of life and gastrointestinal well-being after segmental colo-rectal resection for deep infiltrating endometriosis (ENDO-RESECT QoL). Arch. Gynecol. Obstet..

[14] Roman H., Bubenheim M., Huet E., Bridoux V., Zacharopoulou C., Daraï E., Collinet P., Tuech J.-J. (2018). Conservative surgery versus colorectal resection in deep endometriosis infiltrating the rectum: A randomized trial. Hum. Reprod..

[15] Rindos N.B., Fulcher I.R., Donnellan N.M. (2020). Pain and Quality of Life after Laparoscopic Excision of Endometriosis. J. Minim. Invasive Gynecol..

[16] Roman H., Tuech J.-J., Huet E., Bridoux V., Khalil H., Hennetier C., Bubenheim M., Branduse L.A. (2019). Excision versus colorectal resection in deep endometriosis infiltrating the rectum: 5-year follow-up of patients enrolled in a randomized controlled trial. Hum. Reprod..

[17] Nagin D.S. (2014). Group-Based Trajectory Modeling: An Overview. Ann. Nutr. Metab..

[18] Comptour A., Pereira B., Lambert C., Chauvet P., Gremeau A.-S., Pouly J.-L., Canis M., Bourdel N. (2020). Identification of Predictive Factors in Endometriosis for Improvement in Patient Quality of Life. J. Minim. Invasive Gynecol..

[19] Leplège A., Ecosse E., Verdier A., Perneger T.V. (1998). The French SF-36 Health Survey. J. Clin. Epidemiol..

[20] Stull D.E., Wasiak R., Kreif N., Raluy M., Colligs A., Seitz C., Gerlinger C. (2013). Validation of the SF-36 in patients with endometriosis. Qual. Life Res..

[21] Bourdel N., Alves J., Pickering G., Ramilo I., Roman H., Canis M. (2014). Systematic review of endometriosis pain assessment: How to choose a scale?. Hum. Reprod. Updat..

[22] Bourdel N., Roman H., Mage G., Canis M. (2011). Chirurgie des endométriomes ovariens: De la physiopathologie à la prise en charge pratique pré-, per- et postopératoire. Gynécol. Obstét. Fertil..

[23] Kondo W., Bourdel N., Tamburro S., Cavoli D., Jardon K., Rabischong B., Botchorishvili R., Pouly J., Mage G., Canis M. (2010). Complications after surgery for deeply infiltrating pelvic endometriosis. BJOG: Int. J. Obstet. Gynaecol..

[24] Bourdel N., Cognet S., Canis M., Berdugo O., Botchorishvili R., Rabischong B., Jardon K., Botchorishvilli R. (2015). Laparoscopic Ureteroneocystostomy: Be Prepared!. J. Minim. Invasive Gynecol..

[25] Shah N.H., Hipwell A.E., Stepp S.D., Chang C.-C.H. (2014). Measures of discrimination for latent group-based trajectory models. J. Appl. Stat..

[26] Nagin D.S., Jones B.L., Passos V.L., Tremblay R.E. (2016). Group-based multi-trajectory modeling. Stat. Methods Med. Res..

[27] Nagin D. (2005). Group-Based Modeling of Development.

[28] Feise R.J. (2002). Do multiple outcome measures require p-value adjustment?. BMC Med Res. Methodol..

[29] Nagin D.S., Odgers C.L. (2010). Group-Based Trajectory Modeling in Clinical Research. Annu. Rev. Clin. Psychol..

[30] Kho R.M., Andres M.P., Borrelli G.M., Neto J.S., Zanluchi A., Abrão M.S. (2018). Surgical treatment of different types of endometriosis: Comparison of major society guidelines and preferred clinical algorithms. Best Pr. Res. Clin. Obstet. Gynaecol..

[31] Valentin L., Canis M., Pouly J.L., Lasnier C., Jaffeux P., Aublet-Cuvelier B., Bourdel N. (2017). SF-36 preoperative interest of predicting improvement of quality of life after laparoscopic management of minimal endometriosis. J. Gynecol. Obstet. Hum. Reprod..

[32] Stratton P., Berkley K.J. (2010). Chronic pelvic pain and endometriosis: Translational evidence of the relationship and implications. Hum. Reprod. Updat..

[33] Vercellini P., Trespidi L., De Giorgi O., Cortesi I., Parazzini F., Crosignani P.G. (1996). Endometriosis and pelvic pain: Relation to disease stage and localization. Fertil. Steril..

[34] Bourdel N., Chauvet P., Billone V., Douridas G., Fauconnier A., Gerbaud L., Canis M. (2019). Systematic review of quality of life measures in patients with endometriosis. PLoS ONE.

[35] Schwartz A.S.K., Gross E., Geraedts K., Rauchfuss M., Wölfler M.M., Häberlin F., Von Orelli S., Eberhard M., Imesch P., Imthurn B. (2019). The use of home remedies and complementary health approaches in endometriosis. Reprod. Biomed. Online.

[36] Mira T., Buen M.M., Borges M.G., Yela D.A., Benetti-Pinto C.L. (2018). Systematic review and meta-analysis of complementary treatments for women with symptomatic endometriosis. Int. J. Gynecol. Obstet..

[37] Expectations of Women with Endometriosis: What Information to Deliver? CNGOF-HAS Endometriosis Guidelines. https://pubmed.ncbi.nlm.nih.gov/29530554/.

[38] Jones G., Jenkinson C., Kennedy S. (2004). Evaluating the responsiveness of the Endometriosis Health Profile Questionnaire: The EHP-30. Qual. Life Res..

[39] Chauvet P., Auclair C., Mourgues C., Canis M., Gerbaud L., Bourdel N. (2017). Psychometric properties of the French version of the Endometriosis Health Profile-30, a health-related quality of life instrument. J. Gynecol. Obstet. Hum. Reprod..

