# Supplementary material for: Long-Term Evolution of Quality of Life and Symptoms Following Surgical Treatment for Endometriosis: Different Trajectories for Which Patients?
Source: J Clin Med. 2020 Jul 31;9(8):2461. doi: 10.3390/jcm9082461 (PMC7463511; doi:10.3390/jcm9082461)
Supplement: Supplementary file 1 [file jcm-09-02461-s001.zip › Supplemental table 1.docx]

|  | **Trajectories of chronic pelvic pain** | | | |  | **Trajectories of dysmenorrhea** | | | |  | **Trajectories of dyspareunia** | | | |
| --- | --- | --- | --- | --- | --- | --- | --- | --- | --- | --- | --- | --- | --- | --- |
|  | **1** | **2** | **3** | **p** |  | **1** | **2** | **3** | **p** |  | **1** | **2** | **3** | **p** |
| Age (years) | 34.0 ± 8.1 | 32.8 ± 6.5 | 32.1 ± 6.3 | 0.13 |  | **35.1 ± 8.5** | **31.7 ± 5.9** | **32.0 ± 5.9** | **<0.001** |  | **35.0 ± 7.6** | **32.3 ± 6.9** | **31.5 ± 6.2** | **<0.001** |
| BMI (kg/m^2^) | 22.5 ± 3.9 | 21.8 ± 3.5 | 22.0 ± 3.7 | 0.06 |  | 22.3 ± 4.1 | 21.7 ± 3.4 | 22.2 ± 3.5 | 0.16 |  | **22.5 ± 3.9** | **21.6 ± 3.5** | **22.1 ± 3.6** | **0.01** |
| Tobacco (%) | **27.8** | **38.9** | **45.2** | **<0.001** |  | 32.1 | 37.6 | 40.1 | 0.07 |  | 34.7 | 34.7 | 39.9 | 0.26 |
| Single (%) | 20.6 | 22.8 | 21.1 | 0.77 |  | 20.3 | 20.4 | 23.2 | 0.57 |  | **27.3** | **19.0** | **17.7** | **0.004** |
| Menarche (years) | **12.8 ± 1.5** | **12.9 ± 1.7** | **12.6 ± 1.6** | **0.04** |  | 12.9 ± 1.7 | 12.8 ± 1.5 | 12.7 ± 1.5 | 0.61 |  | 12.8 ± 1.5 | 12.9 ± 1.7 | 12.7 ± 1.6 | 0.30 |
| Infertility antecedents (%) | 45.6 | 39.5 | 39.6 | 0.19 |  | **38.1** | **50.4** | **39.9** | **0.01** |  | 37.4 | 46.9 | 42.4 | 0.07 |
| Endometriosis antecedents (%) | 11.2 | 8.4 | 11.6 | 0.35 |  | 11.9 | 9.2 | 9.6 | 0.51 |  | 10.9 | 11.9 | 8.8 | 0.45 |
| Pregnancy desire (%) | 54.5 | 52.9 | 51.6 | 0.79 |  | **48.3** | **62.1** | **52.0** | **0.006** |  | **45.9** | **59.8** | **55.6** | **0.003** |
| Preoperative medical treatment (%) | **23.8** | **32.9** | **31.2** | **0.02** |  | 30.4 | 26.4 | 29.7 | 0.57 |  | 28.0 | 29.6 | 30.1 | 0.82 |
| Main motive for intervention (%)  Pain  Sterility  Endometriosis  Other | **43.3**  **25.3**  **16.6**  **14.8** | **53.9**  **19.3**  **13.3**  **13.5** | **58.5**  **16.3**  **10.7**  **14.4** | **0.007** |  | **43.7**  **20.1**  **19.8**  **16.5** | **57.9**  **20.4**  **10.4**  **11.3** | **54.4**  **21.2**  **10.5**  **14.0** | **0.001** |  | 46.1  19.7  16.8  17.4 | 52.9  23.1  12.8  11.2 | 55.4  19.8  11.4  13.3 | 0.053 |
| rAFS stage (%)  Stage I: minimal  Stage II: mild  Stage III: moderate  Stage IV: severe | 25.8  30.9  19.5  23.7 | 26.5  21.1  27.1  25.3 | 30.7  26.5  21.6  21.2 | 0.66 |  | 28.4  29.4  19.6  22.6 | 27.4  28.4  18.6  25.6 | 26.6  27.4  22.7  23.3 | 0.88 |  | 26.7  29.1  17.5  26.7 | 30.2  28.5  17.5  23.8 | 26.3  27.5  25.8  20.4 | 0.08 |
| Dysmenorrhea (VAS) | **5.1 ± 3.7** | **6.7 ± 2.9** | **7.0 ± 2.8** | **<0.001** |  | **4.6 ± 3.7** | **6.5 ± 2.8** | **7.4 ± 2.4** | **<0.001** |  | **5.4 ± 3.7** | **6.7 ± 2.9** | **6.7 ± 2.8** | **<0.001** |
| Dyspareunia (VAS) | **4.7 ± 2.6** | **5.5 ± 2.3** | **5.6 ± 2.6** | **0.003** |  | 5.3 ± 2.6 | 5.0 ± 2.5 | 5.5 ± 2.4 | 0.10 |  | **0** | **5.2 ± 2.3** | **5.6 ± 2.4** | **<0.001** |
| Chronic pelvic pain (VAS) | 0 | 5.8 ± 2.4 | 6.2 ± 2.4 | 0.06 |  | 5.9 ± 2.5 | 5.7 ± 2.4 | 6.1 ± 2.4 | 0.44 |  | 6.2 ± 2.7 | 5.9 ± 2.3 | 5.9 ± 2.4 | 0.61 |
| Dyschezia (%) | **19.7** | **31.7** | **36.3** | **<0.001** |  | **19.4** | **28.5** | **36.7** | **<0.001** |  | **17.6** | **36.4** | **34.2** | **<0.001** |
| Nausea (%) | **14.8** | **24.5** | **29.6** | **<0.001** |  | **16.8** | **21.3** | **27.9** | **0.001** |  | **17.6** | **25.2** | **25.3** | **0.02** |
| Rectal bleeding (%) | 2.9 | 4.9 | 5.6 | 0.23 |  | 3.8 | 5.4 | 4.2 | 0.65 |  | **2.0** | **5.8** | **5.7** | **0.02** |
| Constipation (%) | **15.7** | **27.4** | **33.0** | **<0.001** |  | **18.5** | **26.2** | **29.2** | **0.003** |  | **15.9** | **26.4** | **32.1** | **<0.001** |
| Diarrhea (%) | **14.5** | **25.6** | **30.0** | **<0.001** |  | **14.1** | **29.0** | **26.9** | **<0.001** |  | **18.2** | **27.7** | **24.2** | **0.02** |
| Painful urination (%) | 8.3 | 11.8 | 15.0 | 0.08 |  | 7.9 | 10.9 | 14.2 | 0.06 |  | **8.0** | **11.3** | **14.7** | **0.045** |
| Fatigue (%) | **40.9** | **62.0** | **61.1** | **<0.001** |  | **42.1** | **52.9** | **65.1** | **<0.001** |  | **44.9** | **54.5** | **62.8** | **<0.001** |
| Headache (%) | **27.0** | **43.8** | **49.3** | **<0.001** |  | **27.9** | **43.4** | **46.6** | **<0.001** |  | **28.1** | **39.7** | **49.7** | **<0.001** |
| Anxiety (%) | **30.4** | **41.8** | **47.0** | **<0.001** |  | **35.0** | **36.2** | **44.4** | **0.02** |  | **31.8** | **38.0** | **47.0** | **<0.001** |
| Depression (%) | **8.7** | **13.3** | **14.8** | **0.048** |  | 11.2 | 10.0 | 14.0 | 0.28 |  | 9.7 | 11.2 | 14.9 | 0.08 |
| Sleep disorder (%) | **12.8** | **16.4** | **23.0** | **0.003** |  | 13.5 | 16.3 | 20.2 | 0.052 |  | 13.6 | 18.2 | 19.3 | 0.11 |
| PCS | **52 ± 10** | **47 ± 10** | **45 ± 10** | **<0.001** |  | **50 ± 11** | **48 ± 10** | **47 ± 10** | **0.01** |  | **50 ± 10** | **48 ± 10** | **46 ± 11** | **<0.001** |
| MCS | **46 ± 11** | **40 ± 13** | **38 ± 11** | **<0.001** |  | **44 ± 13** | **42 ± 12** | **40 ± 12** | **0.004** |  | **45 ± 13** | **42 ± 12** | **39 ± 12** | **<0.001** |

**Supplemental Table 1: Characteristics of patients from different pelvic pain trajectories: univariate analyses.**
