# Supplementary material for: Long-Term Evolution of Quality of Life and Symptoms Following Surgical Treatment for Endometriosis: Different Trajectories for Which Patients?
Source: J Clin Med. 2020 Jul 31;9(8):2461. doi: 10.3390/jcm9082461 (PMC7463511; doi:10.3390/jcm9082461)
Supplement: Supplementary file 1 [file jcm-09-02461-s001.zip › Supplemental table 2.docx]

|  | **Trajectories of PCS** | | | |  | **Trajectories of MCS** | | | |
| --- | --- | --- | --- | --- | --- | --- | --- | --- | --- |
|  | **1** | **2** | **3** | **p** |  | **1** | **2** | **3** | **p** |
| Age (years) | 33.2 ± 7.0 | 32.7 ± 7.0 | 32.2 ± 8.0 | 0.54 |  | 33.6 ± 7.6 | 32.4 ± 6.4 | 32.4 ± 6.6 | 0.18 |
| BMI (kg/m^2^) | 22.0 ± 3.7 | 22.3 ± 3.8 | 22.4 ± 4.2 | 0.51 |  | 22.2 ± 3.7 | 21.9 ± 3.7 | 22.4 ± 3.9 | 0.25 |
| Tobacco (%) | 34.3 | 40.6 | 45.8 | 0.07 |  | **33.1** | **39.2** | **45.4** | **0.03** |
| Single (%) | 22.0 | 18.3 | 33.3 | 0.06 |  | **18.8** | **22.4** | **31.5** | **0.01** |
| Menarche (years) | **12.8 ± 1.5** | **12.8 ± 1.7** | **12.2 ± 1.4** | **0.007** |  | **12.9 ± 1.7** | **12.8 ± 1.5** | **12.3 ± 1.5** | **0.02** |
| Infertility antecedents (%) | 43.4 | 38.9 | 35.4 | 0.30 |  | 42.7 | 43.1 | 32.4 | 0.11 |
| Endometriosis antecedents (%) | 9.9 | 12.1 | 6.3 | 0.44 |  | 10.1 | 10.6 | 10.3 | 0.98 |
| Pregnancy desire (%) | 54.1 | 51.7 | 47.7 | 0.63 |  | 52.2 | 56.7 | 46.1 | 0.15 |
| Preoperative medical treatment (%) | 28.1 | 32.0 | 27.1 | 0.48 |  | 28.9 | 29.7 | 29.0 | 0.96 |
| Main motive for intervention (%) |  |  |  | **0.001** |  |  |  |  | 0.14 |
| Pain | **46.0** | **61.9** | **62.5** |  |  | 49.1 | 52.9 | 57.4 |  |
| Sterility | **23.6** | **14.7** | **14.6** |  |  | 20.2 | 22.7 | 15.7 |  |
| Endometriosis | **15.6** | **10.1** | **10.4** |  |  | 16.5 | 10.5 | 11.1 |  |
| Other | **14.8** | **13.3** | **12.5** |  |  | 14.2 | 13.9 | 15.7 |  |
| rAFS stage (%) |  |  |  | **0.03** |  |  |  |  | 0.37 |
| Stage I: minimal | **27.0** | **27.8** | **31.9** |  |  | 28.6 | 26.1 | 26.2 |  |
| Stage II: mild | **30.4** | **23.3** | **29.8** |  |  | 29.0 | 27.9 | 26.2 |  |
| Stage III: moderate | **20.4** | **19.6** | **29.8** |  |  | 18.1 | 22.2 | 28.2 |  |
| Stage IV: severe | **22.2** | **29.3** | **8.5** |  |  | 24.3 | 23.7 | 19.4 |  |
| Dysmenorrhea (VAS) | **5.6 ± 3.4** | **7.3 ± 2.6** | **8.1 ± 1.6** | **<0.001** |  | **5.7 ± 3.4** | **6.7 ± 2.9** | **7.0 ± 3.2** | **<0.001** |
| Dyspareunia (VAS) | **5.1 ± 2.5** | **5.7 ± 2.4** | **5.5 ± 2.8** | **0.02** |  | **5.1 ± 2.4** | **5.4 ± 2.5** | **6.0 ± 2.7** | **0.02** |
| Chronic pelvic pain (VAS) | **5.6 ± 2.5** | **6.3 ± 2.4** | **6.7 ± 2.4** | **0.002** |  | 6.0 ± 2.5 | 6.0 ± 2.4 | 6.0 ± 2.4 | 0.99 |
| Dyschezia (%) | **24.5** | **37.1** | **35.4** | **<0.001** |  | **24.5** | **34.9** | **28.7** | **0.005** |
| Nausea (%) | **17.0** | **31.7** | **41.7** | **<0.001** |  | **18.2** | **26.7** | **28.7** | **0.004** |
| Rectal bleeding (%) | **3.3** | **6.8** | **4.2** | **0.049** |  | 3.9 | 4.7 | 5.6 | 0.63 |
| Constipation (%) | **22.2** | **29.9** | **29.2** | **0.04** |  | **20.6** | **27.6** | **35.2** | **0.002** |
| Diarrhea (%) | **18.1** | **32.7** | **29.2** | **<0.001** |  | **19.8** | **27.6** | **22.2** | **0.03** |
| Painful urination (%) | **9.3** | **16.2** | **11.1** | **0.02** |  | **7.4** | **15.1** | **18.7** | **0.001** |
| Fatigue (%) | **46.2** | **67.3** | **83.3** | **<0.001** |  | **45.7** | **62.8** | **66.7** | **<0.001** |
| Headache (%) | **34.0** | **47.5** | **62.5** | **<0.001** |  | **32.4** | **46.8** | **48.1** | **<0.001** |
| Anxiety (%) | 37.3 | 42.1 | 47.9 | 0.17 |  | **26.9** | **50.9** | **60.2** | **<0.001** |
| Depression (%) | **11.3** | **11.2** | **27.1** | **0.005** |  | **5.9** | **16.9** | **25.9** | **<0.001** |
| Sleep disorder (%) | **13.7** | **21.6** | **33.3** | **<0.001** |  | **11.2** | **20.3** | **33.3** | **<0.001** |
| PCS | **54 ± 7** | **38 ± 8** | **36 ± 9** | **<0.001** |  | **49 ± 10** | **48 ± 11** | **47 ± 12** | 0.12 |
| MCS | **42 ± 12** | **42 ± 12** | **35 ± 13** | **<0.001** |  | **50 ± 9** | **34 ± 10** | **29 ± 11** | **<0.001** |

**Supplemental Table 2: Characteristics of patients from different MCS and PCS trajectories: univariate analyses**
