# Supplementary material for: Long-Term Evolution of Quality of Life and Symptoms Following Surgical Treatment for Endometriosis: Different Trajectories for Which Patients?
Source: J Clin Med. 2020 Jul 31;9(8):2461. doi: 10.3390/jcm9082461 (PMC7463511; doi:10.3390/jcm9082461)
Supplement: Supplementary file 1 [file jcm-09-02461-s001.zip › jcm-861530 suppl titles.docx]

Supplemental Figure 1: Variable categories (A) and individual positioning (B) on the two first axes following multiple correspondence analysis (MCA). A) Relationships between variable categories can be interpreted as follows: categories with a similar profile are close to each other, negatively correlated categories are plotted on opposite sides of the origin, the distance between categories and the origin indicates category quality (distant points from the origin are well represented by the MCA). B) Factorial analysis revealed 4 patient profiles prior to surgery.

age29_35: 29 ≤ age < 35; ageInf29: age < 29; ageSup35: age ≥ 35; AnT: analgesic treatment; anx: anxiety; bmi25: BMI < 25; bmiSup25 : BMI ≥ 25; Cpp Inf3: chronic pelvic pain (VAS < 3); Cpp 3_6: chronic pelvic pain (3 ≤ VAS ≤ 6); Cpp Sup7: chronic pelvic pain (VAS ≥ 7); con: constipation; dep: depression; dia: diarrhea; Dhom: discomfort at home; dop: desire of pregnancy; DsoE: discomfort during social event; Dspo: discomfort during sport; Dslee: discomfort during sleep; Dsta: standing discomfort; Dsit: sitting discomfort; Dwal: discomfort during walking; dyInf3: dysmenorrhea (VAS < 3); dy3_6: dysmenorrhea (3 ≤ VAS ≤ 6); dySup7: dysmenorrhea (VAS ≥ 7); dyspaInf3: dyspareunia (VAS < 3); dyspa3_6: dyspareunia (3 ≤ VAS ≤ 6); dyspaInf7: dyspareunia (VAS ≥ 7); dys: dysuria; FEn: family endometriosis antecedent; fat: fatigue; Frus: frustration; gynT: gynecological treatment; hea: headaches; inf: infertility; mcsInf40: MCS ≤ 40; nau: nausea; NbLBC: number of live birth; PaU: painful urination; pcsInf50: PCS ≤ 50; rbl: rectal bleding; sle: sleep disorders; tab: tabacco; stg1,2,3,4: stage 1,2,3 or 4 (rAFS). N: no, Y: yes, DM: during menstruation, 0: never, 1: occasionally, 2: always.

Table 2: Characteristics of the 4 clusters obtained by multiple correspondence analysis. Data are presented as percentages. MCS: mental component summary; PCS: physical component summary; rAFS: revised American fertility society; VAS: visual analog scale.

Table 3: Crossing of the different trajectories with clusters. Cpp: chronic pelvic pain

Figure 4: Characteristics of each cluster of patients. Cpp: chronic pelvic pain; QoL: quality of life

Supplemental Table 1: Characteristics of patients from different pelvic pain trajectories: univariate analyses. Data are presented as percentages or mean ± standard deviation. BMI: body mass index; MCS: mental component summary; PCS: physical component summary; rAFS: revised American fertility society; VAS: visual analog scale.

Supplemental Table 2: Characteristics of patients from different MCS and PCS trajectories: univariate analyses. Data are presented as percentages or mean ± standard deviation. BMI: body mass index; MCS: mental component summary; PCS: physical component summary; rAFS: revised American fertility society; VAS: visual analog scale.
