# Supplementary material for: Long-Term Evolution of Quality of Life and Symptoms Following Surgical Treatment for Endometriosis: Different Trajectories for Which Patients?
Source: J Clin Med. 2020 Jul 31;9(8):2461. doi: 10.3390/jcm9082461 (PMC7463511; doi:10.3390/jcm9082461)
Supplement: Supplementary file 1 [file 23042020_SFigure 1.pdf › 23042020_SFigure 1.pdf]

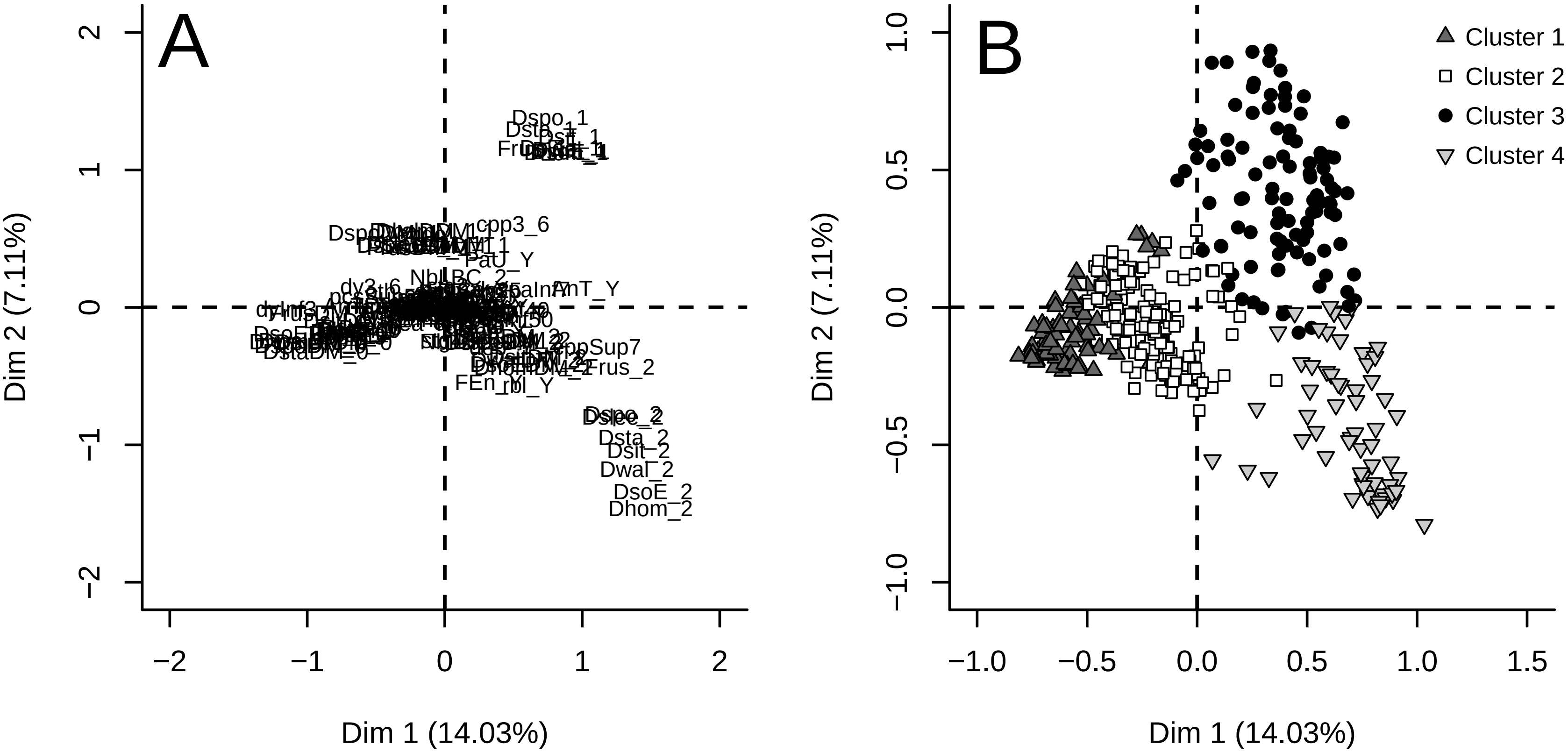
A
B
Dim 2 (7.11%)
Dim 1 (14.03%)
Dim 2 (7.11%)
Dim 1 (14.03%)
2
1
0
−1
−2
−2
−1
0
1
2
1.0
0.5
0.0
−0.5
−1.0
−1.0
−0.5
0.0
0.5
1.0
1.5
Cluster 1
Cluster 2
Cluster 3
Cluster 4
Dspo_1
Dsta_1
cpp3_6
PaU_Y
NbLBC_2
AnT_Y
dy3_6
cppSup7
Frus_2
FEn_Y
rbl_Y
Dspo_2
Dsta_2
Dsit_2
Dwal_2
DsoE_2
Dhom_2
DstaDM_0
